# OpenSHS: Open Smart Home Simulator

**DOI:** 10.3390/s17051003

**Published:** 2017-05-02

**Authors:** Nasser Alshammari, Talal Alshammari, Mohamed Sedky, Justin Champion, Carolin Bauer

**Affiliations:** 1 Staffordshire University, College Road, ST4 2DE Stoke-on-Trent, UK; talal.alshammari@research.staffs.ac.uk (T.A.); m.h.sedky@staffs.ac.uk (M.S.); j.j.champion@staffs.ac.uk (J.C.); c.i.bauer@staffs.ac.uk (C.B.); 2 College of Information and Computer Science, Aljouf University, Sakaka 72388, Saudi Arabia; 3 College of Computer Science and Engineering, University of Hail, Hail 53962, Saudi Arabia

**Keywords:** smart home, simulation, internet of things, machine learning, visualisation

## Abstract

This paper develops a new hybrid, open-source, cross-platform 3D smart home simulator, OpenSHS, for dataset generation. OpenSHS offers an opportunity for researchers in the field of the Internet of Things (IoT) and machine learning to test and evaluate their models. Following a hybrid approach, OpenSHS combines advantages from both interactive and model-based approaches. This approach reduces the time and efforts required to generate simulated smart home datasets. We have designed a replication algorithm for extending and expanding a dataset. A small sample dataset produced, by OpenSHS, can be extended without affecting the logical order of the events. The replication provides a solution for generating large representative smart home datasets. We have built an extensible library of smart devices that facilitates the simulation of current and future smart home environments. Our tool divides the dataset generation process into three distinct phases: first design: the researcher designs the initial virtual environment by building the home, importing smart devices and creating contexts; second, simulation: the participant simulates his/her context-specific events; and third, aggregation: the researcher applies the replication algorithm to generate the final dataset. We conducted a study to assess the ease of use of our tool on the System Usability Scale (SUS).

## 1. Introduction

With the recent rise of the Internet of Things, analysing data captured from smart homes is gaining more research interest. Moreover, developing intelligent machine learning techniques that are able to provide services to the smart home inhabitants are becoming popular research areas.

Intelligent services, such as the classification and recognition of activities of daily living (ADL) and anomaly detection in elderly daily behaviour, require the existence of good datasets that enable testing and validation of the results [1,2,3,4]. The medical field also recognised the importance of analysing ADLs and how these techniques are effective at detecting medical conditions for the patients [5]. These research projects require either real or synthetic datasets that are representative of the scenarios captured from a smart home. However, the cost to build real smart homes and the collection of datasets for such scenarios is expensive and sometimes infeasible for many projects [4,6,7,8,9]. Moreover, several issues face the researchers before actually building the smart home, such as finding the optimal placement of the sensors [10], lack of flexibility [9,11], finding appropriate participants [4,7] and privacy and ethical issues [12].

Even though there exist real smart home datasets [13,14,15], sometimes, they do not meet the needs of the conducted research project; such as the need to add more sensors or to control the type of the generated scenarios. Very few of such datasets record the readings of the sensors in real time and provide a detailed time-stamped field, like the Activity Recognition with Ambient Sensing dataset (ARAS) [14]. Moreover, preparing a real dataset could be a laborious task, and if not done with care, it could lead to producing erroneous output.

When building real smart home test beds, several challenges are facing the preparation of real datasets. One challenge is having a robust and continuous capturing mechanism for the sensors’ data. Another challenge is following an appropriate annotation method for the inhabitants’ activities.

**Figure 1:**
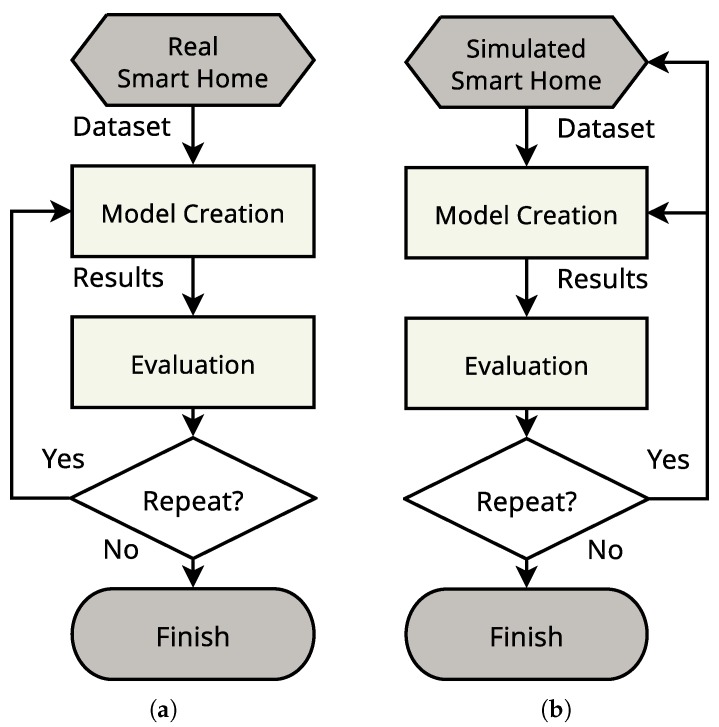
The workflow of real and simulated smart homes test beds. (**a**) A real test bed; (**b**) a simulated test bed.

The existence of a dataset simulation tool overcomes the drawbacks/challenges of generating real datasets. Such tools facilitate fast dataset generation and offer robust methods to capture the sensors’ data. Additionally, they can offer solutions such as the ability to pause and fast-forward the simulation to enable more accurate activity annotation. When developing machine learning models, targeting specific functionalities, researchers rely on the existence of good representative datasets. A common practice in machine learning is to divide the dataset into two parts, training and testing. The model creation starts by initialising its parameters and training on a portion of the dataset. Then, the model will be tested on another part of the same dataset, and its results will be evaluated. The results of the evaluation could reveal the need to redesign the smart home by adding or removing smart devices or changing the scenarios generated, etc. In the case of a real smart home, if the results revealed the need to change something, this is usually a costly and infeasible choice to make. Therefore, the researcher could only be able to tweak the model parameters as shown in Figure 1a. On the other hand, with a simulated smart home, this can be easily done, and the researcher can go back and modify the smart home design as shown in Figure 1b.

The approaches for the smart home simulation tools can be divided into model-based and interactive approaches. The model-based approaches use statistical models to generate datasets, while the interactive approaches rely on real-time capturing of fine-grained activities using an avatar controlled by a human/simulated participant. Each approach has its advantages and disadvantages.

From what we mentioned earlier, it is apparent that the virtual simulation tool should offer far greater flexibility and lower cost than conducting an actual and physical smart home simulation [6]. The new recent advances in computer graphics, such as virtual reality (VR) technologies, can provide immersive and semi-realistic experiences that could come close to the real experience. The simulation tool should also be open and readily available to both the researchers and the test subjects.

Although there are some research efforts available in the literature for smart home simulation tools, they suffer from some limitations. The majority of these tools are not available in the public domain as an open-source project or limited to a particular platform. Furthermore, most of the publicly-available simulation tools lack the flexibility to add and customise new sensors or devices.

When generating datasets, the model-based approaches are capable of generating bigger datasets, but the granularity of captured interactions is not as fine as the interactive approaches. However, the interactive approaches usually take a longer time to produce the datasets, as they capture the interactions in real time.

In this paper, we present the architecture and implementation of OpenSHS, a novel smart home simulation tool. OpenSHS is a new hybrid, open-source, cross-platform 3D smart home simulator for dataset generation. Its significant contribution is that OpenSHS offers an opportunity for researchers in the field of the Internet of Things (IoT) and machine learning to produce and share their smart home datasets, as well as testing, comparing and evaluating their models objectively. Following a hybrid approach, OpenSHS combines advantages from both interactive and model-based approaches. This approach reduces the time and efforts required to generate simulated smart home datasets. OpenSHS includes an extensible library of smart devices that facilitates the simulation of current and future smart home environments. We have designed a replication algorithm for extending and expanding a dataset. A small sample dataset produced, by OpenSHS, can be extended without affecting the logical order of the events. The replication provides a solution for generating large representative smart home datasets. Moreover, OpenSHS offers a feature for shortening and extending the duration of the generated activities.

The rest of this paper is structured as follows: The following section reviews existing real smart home test beds and simulation tools; this section is concluded by analysing existing smart home simulation tools and comparing them with our proposed tool, OpenSHS. Section 3 presents the architecture of OpenSHS and its implementation. Section 4 presents two usability studies for using OpenSHS by researchers and participants. Followed by Section 5, which lists the limitations of OpenSHS and the planned future work for this project, the paper concludes.

## 2. Related Work

The literature is rich with efforts that focus on generating datasets for smart home applications. These efforts can be classified into two main categories, datasets generated either from real smart homes test beds or using smart home simulation tools.

### 2.1. Real Smart Home Test Beds

One of the recent projects for building real smart homes for research purposes was the work carried out by the Centre for Advanced Studies in Adaptive Systems (CASAS) [16], where they created a toolkit called ‘smart home in a box’, which is easily installed in a home to make it able to provide smart services. The components of the toolkit are small and can fit in a single box. The toolkit has been installed in 32 homes to capture the participants’ interactions. The datasets are publicly available online [17].

The TigerPlace [18] project is an effort to tackle the growing ageing population. Using passive sensor networks implemented in 17 apartments within an elder-care establishment. The sensors include motion sensors, proximity sensors, pressure sensors and other types. The data collection took more than two years for some of the test beds.

SmartLab [19] is a smart laboratory devised to conduct experiments in smart living environments to assess the development of independent living technologies. The laboratory has many types of sensors, such as pressure, passive infrared (PIR) and contact sensors. The participants’ interactions with SmartLab are captured in an XML-based schema called homeML [20].

The Ubiquitous Home [21] is a smart home that was built to study context-aware services by providing cameras, microphones, pressure sensors, accelerometers and other sensor technologies. The home consists of several rooms equipped with different sensors. To provide contextual services to each resident, the Ubiquitous Home recognises the resident by providing radio-frequency identification (RFID) tags and by utilising the installed cameras.

PlaceLab [22] is a 1000 sq. ft.smart apartment that has several rooms. The apartment has many sensors distributed throughout each room, such as electrical current sensors, humidity sensors, light sensors, water flow sensors, etc. Volunteering participant can live in PlaceLab to generate a dataset of their interaction and behaviour. The project produced several datasets for different scenarios [23].

HomeLab [24] is a smart home equipped with 34 cameras distributed around several rooms. The project has an observation room that allows the researcher to observe and monitor the conducted experiments. HomeLab aims to provide datasets to study human behaviour in smart environments and investigate technology acceptance and usability.

The GatorTech smart home [25] is a programmable and customisable smart home that focuses on studying the ability of pervasive computing systems to evolve and adapt to future advances in sensor technology.

### 2.2. Smart Home Simulation Tools

Smart home simulation tools can be categorised into two main approaches, according to Synnott et al. [6]: model-based and interactive approaches.

#### 2.2.1. Model-Based Approach

This approach uses pre-defined models of activities to generate synthetic data. These models specify the order of events, the probability of their occurrence and the duration of each activity. This approach facilitates the generation of large datasets in a short period. However, the downside of this approach is that it cannot capture intricate interactions or unexpected accidents that are common in real homes. An example of such an approach is the work done by Mendez-Vazquez et al. [7].

PerSim 3D [26] is a tool to simulate and model user activities in smart spaces. The aim of this tool is to generate realistic datasets for complex scenarios of the inhabitant’s activities. The tool provides a graphical user interface (GUI) for visualising the activities in 3D. The researcher can define contexts and set ranges of acceptable values for the sensors in the smart home. However, the tool is not available freely in the public domain.

SIMACT [27] is a 3D smart home simulator designed for activity recognition. SIMACT has many pre-recorded scenarios that were captured from clinical experiments, which can be used to generate datasets for the recognition of ADLs. SIMACT is a 3D open-source and cross-platform project developed with Java and uses the Java Monkey Engine (JME) [28] as its 3D engine.

DiaSim [29] is a simulator developed using Java for pervasive computing systems that can deal with heterogeneous smart home devices. It has a scenario editor that allows the researcher to build the virtual environment to simulate a certain scenario.

The Context-Aware Simulation System (CASS) [30] is another tool that aims at generating context information and testing context-awareness applications in a virtual smart home. CASS allows the researcher to set rules for different contexts. A rule can be, for example, turn the air conditioner on if a room reaches a specific temperature. The tool can detect conflicts between the rules of the pre-defined contextual scenarios and determine the best positioning of the sensors. CASS provides a 2D visualisation GUI for the virtual smart home.

The Context-Awareness Simulation Toolkit (CAST) [31] is a simulation tool designed to test context-awareness applications and provides visualisations of different contexts. The tool generates context information from the users in a virtual smart home. CAST was developed with the proprietary technology Adobe Flash and is not available in the public domain.

#### 2.2.2. Interactive Approach

Contrary to the previous approach, the interactive approach can capture more interesting interactions and fine details. This approach relies on having an avatar that can be controlled by a researcher, human participant or simulated participant. The avatar moves and interacts with the virtual environment, which has virtual sensors and/or actuators. The interactions could be done passively or actively. One example of passive interactions is having a virtual pressure sensor installed on the floor, and when the avatar walks on it, the sensor should detect this and emit a signal. Active interactions involve actions such as opening a door or turning the light on or off. The disadvantage of this approach, however, is that it is a time-consuming approach to generate sufficient datasets, as all interactions must be captured in real time.

Park et al. [32] presented a virtual space simulator that can generate inhabitants’ data for classifications problems. In order to model inhabitant activities in 3D, the simulator was built using Unity3D [33].

The Intelligent Environment Simulation (IE Sim) [34] is a tool used to generate simulated datasets that capture normal and abnormal ADLs of inhabitants. It allows the researcher to design smart homes by providing a 2D graphical top-view of the floor plan. The researcher can add different types of sensors such as temperature sensors, pressure sensors, etc. Then, using an avatar, the simulation can be conducted to capture ADLs. The format of the generated dataset is homeML [20]. To the knowledge of the authors, IE Sim is not available in the public domain.

Ariani et al. [35] developed a smart home simulation tool that uses ambient sensors to capture the interactions of the inhabitants. The tool has a map editor that allows the researcher to design a floor plan for a smart home by drawing shapes on a 2D canvas. Then, the researcher can add ambient sensors to the virtual home. The tool can simulate binary motion detectors and binary pressure sensors. To simulate the activities and interactions in the smart home, they used the A* pathfinding algorithm [36], to simulate the movement of the inhabitants. During the simulation, all interactions are sampled at 5 Hz and stored into an XML file.

UbiREAL [37] is a Java-based simulation tool that allows the development of ubiquitous applications in a 3D virtual smart space. It allows the researcher to simulate the operations and communications of the smart devices at the network level.

V-PlaceSims [38] is a simulation tool that allows a smart home designer to design a smart home from a floor plan. Then, it allows multiple users to interact with this environment through a web interface. The focus of this tool is the improvement of the designs and management of the smart home.

In addition to the above outlined simulation tools, there are other commercial simulation tools targeting the industry, such as [39,40,41].

Generally, the model-based approach allows the researcher to generate large datasets in short simulation time, but sacrifices the granularity of capturing realistic interactions. On the other hand, the interactive approach captures these realistic interactions, but sacrifices the short and quick simulation time, and therefore, the generated datasets are usually smaller than the ones generated by the model-based approach.

### 2.3. Analysis

Synnott et al. [6] identified several challenges that face the smart home simulation research. One of the key challenges is that many of the available simulation tools [9,11,30,37,38,42,43,44] focus on testing applications that provide context awareness and visualisation rather than focusing on generating representative datasets. Few of the available tools focus on generating datasets [1,12,45,46]. Another key challenge is to have the flexibility and scalability to add new/customised types of smart devices, change their generated output(s), change their positions within the smart home, etc. The multiple inhabitants’ support is also one of the limitations facing the currently-available tools, as this feature is known to be difficult to implement [6].

The review of available smart home simulation tools reveals that the majority of the reported work lacks the openness and availability of the software implementation, which hinders their benefit to the wider research community. Moreover, less than half of the reviewed tools (10 out of 23) do not support multiple operating systems, which can be an issue when working with research teams and/or test subjects. Table 1 shows the analysis and comparison of our proposed tool, OpenSHS, with the existing simulation tools. SIMACT [27] and UbiWise [44] were the only open-source and cross-platform simulation tools available; however, the data generation approach used in that tool is based on a pre-defined script that the researcher plays back within the 3D simulation view.

Apart from the work by [47], this analysis shows that none of the reviewed simulation tools follows a hybrid approach, i.e., a tool that combines the ability of model-based tools to generate large datasets in a reasonable time while keeping the fine-grained interactions that are exhibited by the interactive tools.

Our review shows that fewer simulation tools focus on generating datasets, while the majority of the reviewed tools focus on visualisation and context-awareness applications.

Supporting the simulation of multiple inhabitants is a tricky task, especially for the tools that focus of generating datasets. Most of these tools have an avatar controlled by a single participant at a given time. To have multiple participants conducting a simulation at the same time is one of the identified challenges.

When comparing OpenSHS against the available simulation tools reviewed in Table 1, unlike the majority of such tools, our tool is based on Blender and Python, which are open-source and cross-platform solutions; this offers the following benefits:

- Improving the quality of the state of the art datasets by allowing the scientific community to openly converge on standard datasets for different domains,
- Easier collaborations between research teams from around the globe,
- Faster developments and lower entry barriers,
- Facilitates the objective evaluations and assessments.

Our tool allows the simulations to be conducted in 3D from a first-person perspective. The only open-source tools we could identify in the literature were SIMACT [27,44]. However, neither of these tools focuses on generating datasets. SIMACT does not allow the participant to create specialised simulations. Instead, it relies on pre-recorded data captured from clinical trials.

IE Sim [34] was extended to use a probabilistic model (Poisson distribution) to augment the interactively recorded data by IE Sim. Therefore, the extended version of IE Sim uses a hybrid approach. However, IE Sim is a 2D simulator, which takes part of the realism out of the simulation. This might be a problem when 3D motion data are important to the researcher, for example in anomaly detection algorithms, as identified by [47].

The fast-forwarding feature makes the simulation less cumbersome especially when the simulation has long periods of inactivity, as in elder-care research. This feature is relevant to interactive and hybrid approaches. OpenSHS’s fast-forwarding mechanism streamlines the performance of the simulation and allows the participant to skip in time while conducting a simulation.

Although, OpenSHS currently supports the simulation of one smart home inhabitant, however multiple inhabitants’ simulations are partially supported. The current implementation of this feature does not allow real-time simulation of multiple inhabitants. Instead, The first inhabitant records his/her activities, and then, the second inhabitant can start another simulation. The second inhabitant will be able to see the first inhabitant’s actions played back in the virtual environment.

The approach that OpenSHS uses to generate datasets can be thought of as a middle ground between the model-based and interactive approaches. The replication mechanism that OpenSHS adapts allows for a quick dataset generation, similar to the model-based approaches. Moreover, the replications have richer details, as the activities are captured in real time, similar to the interactive approaches. Overall, the advantages of OpenSHS can be summarised as follows:

1. Accessibility: The underlying technologies used to develop OpenSHS allowed it to work on multiple platforms, thus ensuring a better accessibility for the researchers and the participants alike.
2. Flexibility: OpenSHS gives the researchers the flexibility to simulate different scenarios according to their needs, by adding and/or removing sensors and smart devices. OpenSHS can be easily modified and customised in terms of positioning and changing the behaviour of the smart devices in the virtual smart home to meet the needs of a research project.
3. Interactivity: Capturing the interactions between the participant and the smart home in OpenSHS was done in a real-time fashion, which facilitates the generation of richer datasets.
4. Scalability: Our simulation tool is scalable and easily extensible to add new types of smart devices and sensors. OpenSHS has a library of smart devices that we will keep developing and updating as new types of smart devices become available.
5. Reproducibility: By being an open-source project, OpenSHS does have the advantage of facilitating reproducibility and allowing research teams to produce datasets to validate other research activities.

## 3. OpenSHS Architecture and Implementation

This paper proposes a new hybrid, open-source and cross-platform 3D smart home simulation tool for dataset generation, OpenSHS [51], which is downloadable from http://www.openshs.org under the GPLv2 license [52]. OpenSHS tries to provide a solution to the issues and challenges identified by Synnott et al. [6]. OpenSHS follows a hybrid approach, to generate datasets, combining the advantages of both model-based and interactive approaches. This section presents the architecture of OpenSHS and the technical details of its implementation, which is based on Blender [53] and Python. In this section, we will refer to two entities, the researcher and the participant. The researcher is responsible for most of the work with OpenSHS. The participant is any person volunteering to simulate their activities.

Working with OpenSHS can be divided into three main phases: design phase, simulation phase and aggregation phase. The following subsections will describe each phase.

### 3.1. Design Phase

**Figure 2:**
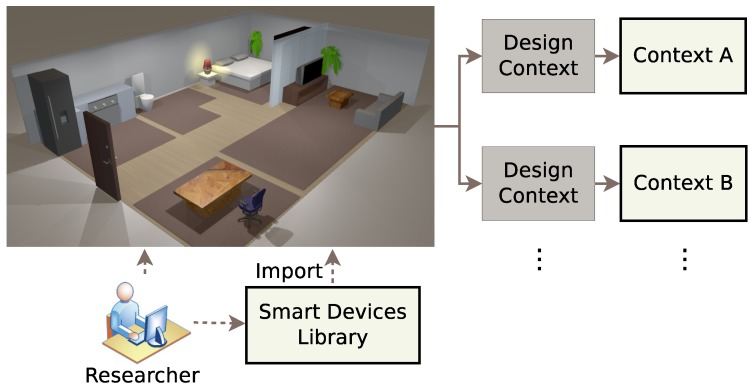
The design phase.

In this phase, as shown in Figure 2, the researcher builds the virtual environment, imports the smart devices, assigns activities’ labels and designs the contexts.

#### 3.1.1. Designing Floor Plan

The researcher designs the 3D floor plan by using Blender, which allows the researcher to easily model the house architecture and control different aspects, such as the dimensions and the square footage. In this step, the number of rooms and the overall architecture of the home are defined according to the requirements of the experiment.

#### 3.1.2. Importing Smart Devices

After the design of the floor plan, the smart devices can be imported into the smart home from the smart devices library, offered by OpenSHS. The current version of OpenSHS includes the following list of active and passive devices/sensors:

- Pressure sensors (e.g., activated carpet, bed, couch, etc.),
- Door sensors,
- Lock devices,
- Appliance switches (TV, oven, fridge, etc.),
- Light controllers.

The smart devices library is designed to be a repository of different types of smart devices and sensors. This list is extensible, as it is programmed with Python. Moreover, the researcher can build a customised sensor/device.

#### 3.1.3. Assigning Activity Labels

OpenSHS enables the researcher to define an unlimited number of activity labels. The researcher decides how many labels are needed according to the experiment’s requirements. Figure 4 shows a prototype where the researcher identified five labels; namely, ‘sleep’, ‘eat’, ‘personal’, ‘work’ and ‘other’. This list of activity labels represents a sample of activities, which the researchers can tailor to their needs.

#### 3.1.4. Designing Contexts

After designing the smart home model, the researcher designs the contexts to be simulated. The contexts are specific time frames that the researcher is interested in simulating, e.g., morning, afternoon, evening contexts. For instance, if the researcher aims to simulate the activities that a participant performs when he/she comes back from work during a weekday, then the researcher will design a context for that period. Finally, the researcher specifies the initial states of the devices for each context.

### 3.2. Simulation Phase

**Figure 3:**
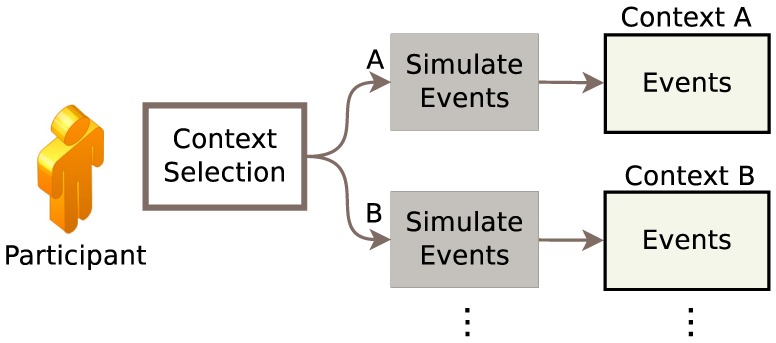
The simulation phase.

Figure 3 shows the overall architecture of the simulation phase. The researcher starts the tool from the OpenSHS interface module, which allows the researcher to specify which context to simulate. Each context has a default starting date and time, and the researcher can adjust the date and time if he/she wants. Every context has a default state for the sensors and the 3D position of the avatar. Then, the participant starts simulating his/her ADLs in that context. During the simulation time, the sensors’ outputs and the state of different devices are captured and stored in a temporary dataset. OpenSHS adapts a sampling rate of one second by default, which the researcher can re-configure as required. Once the participant finishes a simulation, the application control is sent back to the main module to start the simulation of another context.

The simulation phase aims to capture the granularity of the participants’ realistic interactions. However, capturing these fine-grained activities in extended periods of time adds a burden on the participant(s) and sometimes becomes infeasible. OpenSHS offers a solution that mitigates this issue by adapting a fast-forwarding mechanism.

#### 3.2.1. Fast-Forwarding

**Figure 4:**
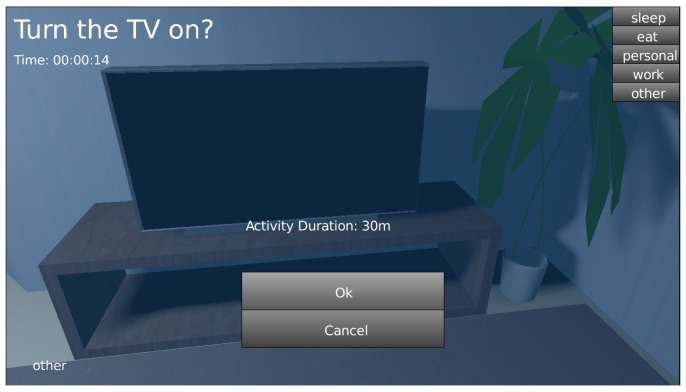
The activity selection and fast-forwarding dialogue.

OpenSHS allows the participant to control the time span of a certain activity: fast-forwarding. For example, if the participant wants to watch the TV for a period of time and does not want to perform the whole activity in real time (since there are no changes in the readings of the home’s sensors), the participant can initiate that activity and spawn a dialogue to specify how long this activity lasts. This feature allows the simulation process to be quick and streamlined. The tool will simply copy and repeat the existing state of all sensors and devices during the specified time period. Figure 4 shows the activity fast-forwarding dialogue during a simulation.

#### 3.2.2. Activities Labelling

The researcher is responsible for familiarising the participant with the available activity labels to choose from. During a simulation and before transitioning from one activity to another, the participant will spawn the activity dialogue shown in Figure 4 to choose the new activity from the available list. To ensure a clean transition from one activity to another, OpenSHS will not commit the new label at the exact moment of choosing the new label. Instead, the new label will be committed when a sensor changes its state. For example, in Figure 6, the transition from the first activity (sleep) to the second (personal) is committed to the dataset when the sensor bedroomLightchanges its state, even though the participant did change the label a couple of seconds earlier.

### 3.3. Aggregation Phase

**Figure 5:**
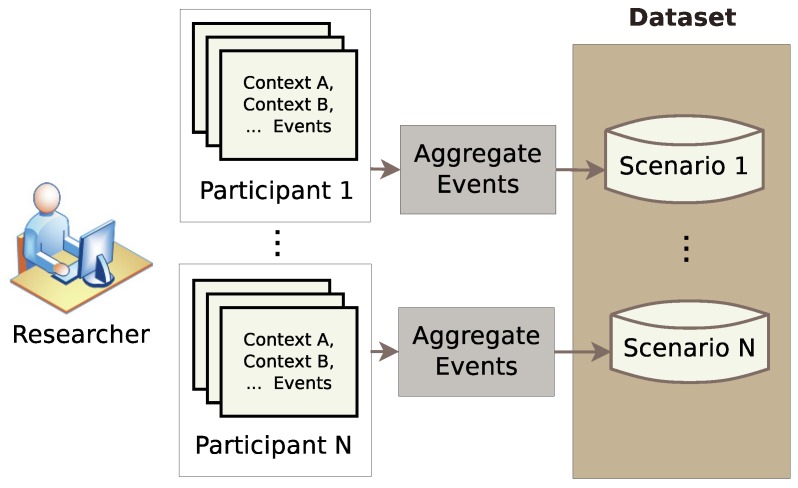
The aggregation phase.

After performing the simulation by the participants, the researcher can aggregate the participants’ generated sample activities, i.e., events, in order to produce the final dataset. The results of the simulation phase forms a pool of sample activities for each context. The aggregation phase aims to provide a solution for the generation of large datasets in short simulation time as shown in Figure 5. Hence, this work develops an algorithm that replicates the output of the simulation phase by drawing appropriate samples for each designated context.

This feature encapsulates the model-based approach’s advantage with the interactive approach adapted by the simulation phase, which allows OpenSHS to combine the benefits of both approaches, a hybrid approach.

#### 3.3.1. Events Replication

It was evident from the beginning of the development of this project that it is not feasible for a participant to sit down and simulate his/her ADLs for a whole day. Moreover, we wanted to capture the interactions between the inhabitant and the smart home in real time. At the same time, we wanted the process to be less tedious and as streamlined as possible. These requirements brought up the concept of real-time context simulations. Instead of having the user simulating his/her ADLs for extended periods of time, the user simulates only a particular context in real time. For example, let us assume we are interested in an ‘early morning’ context, and we want to capture the activities that the inhabitant is doing in this time frame, such as what is usually done in the weekdays compared to the weekends in the same context (the ‘early morning’ context). The user will only perform sample simulations of different events in real time. The greater the number of samples simulated, the richer the generated dataset will be.

To gain more insight into how OpenSHS works, we have built a virtual smart home environment consisting of a bedroom, a living room, a bathroom, a kitchen and an office. Each room is equipped with several sensors totalling twenty-nine sensors of different types. The sensors are binary, and they are either on or off at any given time step.

**Figure 6:**
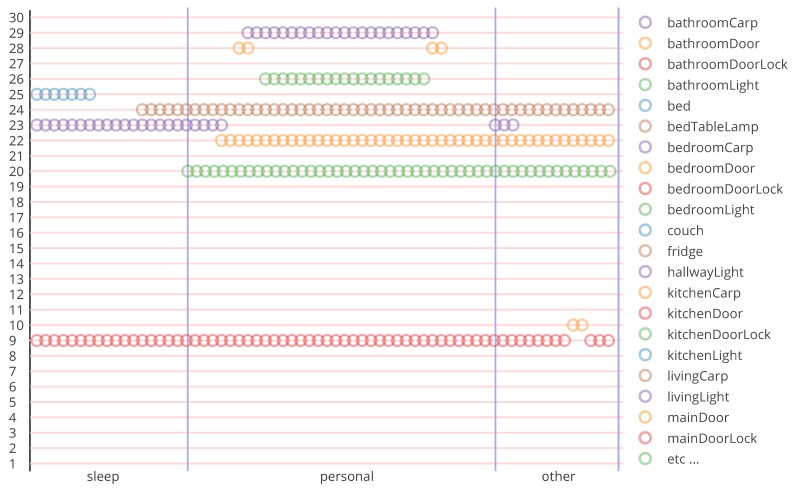
Twenty-nine binary sensors’ output and the corresponding activity labels.

The result of performing a context simulation can be illustrated by Figure 6. The sample consists of three activity labels, namely ‘sleep’, ‘personal’ and ‘other’. Each activity label corresponds to a set of sensors’ readings. The sensors’ readings in the previous figure are readings of binary sensors, and the small circles correspond to an ‘ON-state’ of that sensor.

It is not realistic to aggregate the final dataset by trivially duplicating the contexts samples. There is a need for an algorithm that can replicate the recorded samples to generate a larger dataset. We have designed a replication algorithm for extending and expanding the recorded samples. A small number of simulated events can be extended without affecting their logical order.

To explain the replication algorithm, it is best illustrated by an example. Table 2 shows a set of five samples with their activity labels for a certain context. The first sample has five activities, the second sample has three activities, and so on. When the researcher aggregates the final dataset, the samples of every context are grouped by the number of activities in each sample. Therefore, for the previous example, Sample 1 will be in one group; Samples 2 and 3 will be in a second group; and Samples 4 and 5 will be in a third group. Then, a random group will be chosen, and from that group, a sample will be drawn for each activity. For example, let us take the second group, which contains Samples 2 and 3. The number of activities in this group is three. Therefore, for the first activity, we will either pick the ‘sleep’ activity from Sample 2 or the ‘sleep’ activity from Sample 3. The same procedure will be done for the second and third activities. The output will resemble what is shown in Table 3.

The context samples shown in Table 2 will produce 25 unique replicated copies. In general, the number of unique replicated copies for a single context can be calculated by the Equation (1). Let G denote the number of the groups of unique length of activities; let $S_{g}$ denote the number of samples for the group *g*; and let A denote the number of activities within a sample $S_{g}$. The total number of unique replicated copies R is:

(1) $$R=\sum_{g=1}^{G}S_{g}^{A}$$

OpenSHS can modify the original duration of a performed activity by shortening and/or expanding it. To preserve the structure of a certain activity, we look for the longest steady and unchanged sequence of readings. Then, our algorithm randomly chooses a new duration for this sequence. The new modified sequence length can vary between 5% of the original sequence length, up to its full length. The researcher can use this feature by passing the variable-activities option to the aggregation parameters, as will be shown next.

The researcher can configure a number of parameters to control the generated output, such as:

- days: the number of days to be generated;
- start-date: specifies the starting date for the dataset;
- time-margin: the variability of the starting time for the replicated events; for example, assuming we have a sample that was recorded at 7:30 a.m. and we specified the time margin to be 10 min; the replicated sample could start any time from 7:25 a.m. up to 7:35 a.m.;
- variable-activities: make the duration for each activity variable.

#### 3.3.2. Dataset Generation

After running the aggregation algorithm, the researcher can combine all of the scenarios, generated by different participants, into one final comma separated values (CSV) dataset output. Table 4 shows a sample generated dataset.

The time-margin parameter does add a level of sophistication to the timing of the recorded activities. This is useful for applications that rely heavily on the time dimension of activities, for example in anomaly detection research.

### 3.4. Implementation

OpenSHS implementation relies on Blender and its game engine. Blender’s game engine is programmable by Python.

#### 3.4.1. Blender

Blender was chosen to build the majority of the simulation tool and to act as an infrastructure for OpenSHS. The reasons for this choice can be summarised as:

- Open-source: Blender is an open-source 3D modelling and animation software and an actively-developed project by the open-source community. It allows the user to create 3D models and visual effects. The game engine component of Blender allows the user to build complex 3D interactive games and script them with Python, which is an important feature for OpenSHS.
- Cross-platform: Blender is available for the three major operating systems; namely, GNU/Linux, Microsoft Windows and Apple macOS. Blender uses OpenGL [54] for its game engine, which is also a cross-platform 3D technology available for the major operating systems.
- The Blender game engine: Blender’s game engine allowed us to add the interactivity to the simulations. The physics engine facilitates the simulation of different types of real sensors and devices. For example, blender has a ‘near’ sensor, which will only be activated when the 3D avatar controlled by the user is physically near other objects in the scene. Therefore, such a sensor could be used to simulate a proximity sensor easily.

#### 3.4.2. Python

**Figure 7:**
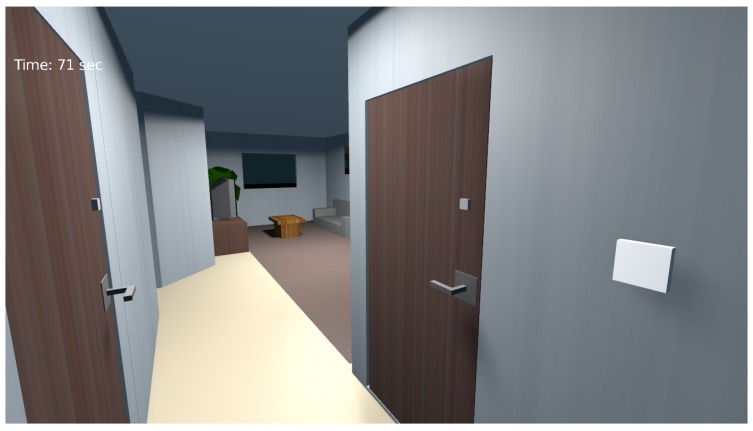
Navigating the smart home space through the first-person perspective.

The interaction with the simulation tool is done by controlling a 3D avatar that navigates the smart home space through a first-person perspective similar to most first-person games. Figure 7 shows the 3D avatar navigating the living room. Since Blender’s game engine uses Python as a programming language, we developed all of the logic and interactions between the avatar and the virtual environment with it. Moreover, all of the OpenSHS modules are programmed by Python.

## 4. OpenSHS Usability

Measuring the usability of a software tool is a challenging and tricky task, since it involves subjective qualities and depends on the context used. John Brooke [55] defines it as “The general quality of the appropriateness to a purpose of any particular artefact”. He developed the widely-used System Usability Scale (SUS), which is a questionnaire consisting of ten questions that measure various aspects of the usability of a system. The score of SUS ranges from 0–100.

To assess OpenSHS usability, we conducted a usability study using SUS. Our sample consists of graduate students and researchers interested in smart home research. We carried out multiple sessions, and in each session, we started by introducing OpenSHS and then by presenting its functionalities. After that, we answered any questions the participants had in mind. Afterwards, we allowed the participants to use OpenSHS and explore its features. Finally, the participants were asked to answer a few questions, such as how frequently do they use their computer on daily basis and whether they play first-person 3D video games or not. Then, the participants were asked to fill out the SUS questionnaire.

We carried out two usability studies: one from the perspective of the researchers and the other from the perspective of the participants using OpenSHS. The researchers’ group was asked to evaluate OpenSHS usability throughout the three phases (design, simulation, aggregation). The participants group was only requested to evaluate the simulation phase.

For the researchers’ group, we collected data from 14 researchers: 85.7% were male and 14.3% female. The average age of the researchers was 36 ($min_{age}=31,max_{age}=43$). All of the researchers reported that they do use their computers on a daily basis, and 93% of them did play 3D first-person games. The aspects that the SUS questionnaire investigates can be summarised as:

1. Frequent use (FU): I think that I would like to use this system frequently.
2. System complexity (SC): I found the system unnecessarily complex.
3. Ease of use (EU): I thought the system was easy to use.
4. Need for support (NS): I think that I would need the support of a technical person to be able to use this system.
5. System’s functions integration (FI): I found the various functions in this system were well integrated.
6. System inconsistencies (SI): I thought there was too much inconsistency in this system.
7. Learning curve (LC): I would imagine that most people would learn to use this system very quickly.
8. How cumbersome the system is (CU): I found the system very cumbersome to use.
9. Confidence in the system (CO): I felt very confident using the system.
10. Need for training before use (NT): I needed to learn a lot of things before I could get going with this system.

**Figure 8:**
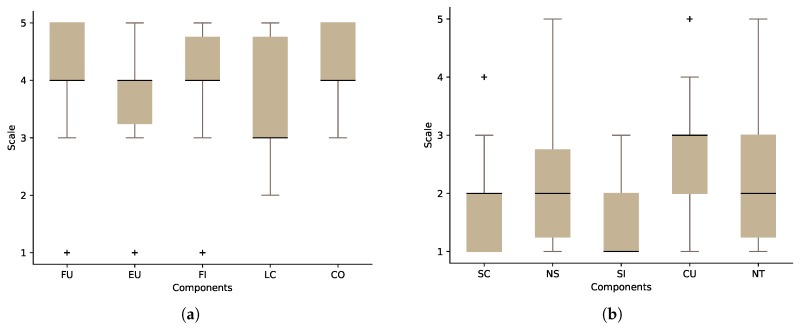
The result of System Usability Scale (SUS) questionnaire for the researchers’ group. (**a**) The positive components; (**b**) the negative components.

Figure 8 shows the results of our SUS questionnaire for the researchers’ group. The odd-numbered statements contribute positively to the overall score if the participant agrees with them (Figure 8a). On the other hand, the even-numbered statements contribute negatively if the researcher agrees with them (Figure 8b). Calculating the score of our sample revealed that the average SUS score of OpenSHS is 71.25 out of 100 ($score_{min}=40,score_{max}=85$).

**Figure 9:**
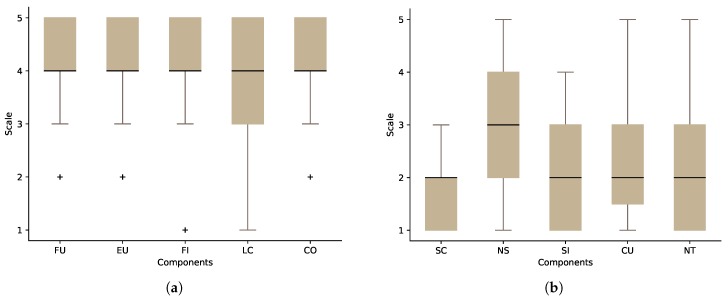
The result of System Usability Scale (SUS) questionnaire for the participants group. (**a**) The positive components; (**b**) the negative components.

For the participants’ group, 31 participants were asked to answer the SUS questionnaire: 77.5% were male, 22.5% female, and the average age of the participants was 27 ($min_{age}=21,max_{age}=36$). Ninety seven percent did play first-person games, and all of the participants reported that they use their computers on a daily basis. Figure 9 shows the participants’ group results. The SUS score for this group is 72.66 out of 100 ($score_{min}=50,score_{max}=87$).

The usability results for both groups are promising, but at the same time, they indicate that there is room for improvements. Both groups agree that the learning curve (LC) component of the questionnaire needs improvement. The results also show the need for support from a technical person to use the system.

## 5. Future Work

For future work, we plan to include full multiple inhabitants support in real time. Moreover, the smart devices library has few specialised sensors that will be updated in the future to include new types of sensors and devices. Another feature that could improve the design phase of the smart home is the addition of a floor plan editor. Taking into consideration that OpenSHS is an open-source project, released under a free and permissive license, the project could envisage quick and rapid development that would facilitate the support of the aforementioned features.

The more realistic the simulation is, the less the need for building actual smart homes to carry out research. Following the growing advancements in computer graphics, virtual reality (VR) is becoming more accessible and affordable. BlenderVR [56] is an open-source framework that extends Blender and allows it to produce immersive and realistic simulations. Since OpenSHS is based on Blender, one of our future goals is to investigate the incorporation of BlenderVR into our tool to provide more true to life experiences for the smart home simulation and visualisation. In terms of accessibility, we aim to make OpenSHS as accessible as possible. Nowadays, the web technologies and web browsers can be a good platform to facilitate the wider distribution of OpenSHS. Technologies such as WebGL [57] can be used to run OpenSHS in different web browsers, and Blender can export to these technologies.

Currently, the labelling of activities is performed by the participant during the simulation phase. OpenSHS does not perform automatic recognition of these activities. As part of our future work, we plan to investigate the possibility of adding automatic recognition of the participants’ activities.

## 6. Conclusions

Many smart home research projects require the existence of representative datasets for their respective applications and research interests and to evaluate and validate their results. Many simulation tools available in the literature focus on context-awareness, and few tools have set dataset generation as their aim. Moreover, there is a lack of open-source simulation tools in the public domain. We developed OpenSHS, an open-source, 3D and cross-platform simulation tool for smart home dataset generation. OpenSHS has many features that allow the researchers to easily design different scenarios and produce highly intricate and representative datasets. Our tool offers a library of smart sensors and devices that can be expanded to include future emerging technologies.

OpenSHS allows the researchers to generate seeds of events rapidly. We have presented a replication algorithm that can extend the simulated events to generate multiple unique large datasets. Moreover, conducting a simulation with a participant can be done in a reasonable time, and we provided tools that streamline the process, such as fast-forwarding.

Our tool divides the dataset generation process into three distinct phases, design, simulation and aggregation. In the design phase, the researcher creates the initial virtual environment by building the home, importing smart devices and creating contexts. In the simulation phase, the participant uses the virtual home to generate context-specific events. In the final stage, the researcher applies the replication algorithm to generate the aggregated dataset.

We conducted a usability study using the System Usability Scale (SUS) to assess how usable OpenSHS is. The results of this study were promising, yet they left room for more improvements.

One of the identified issues in smart home simulations tools is having support for multiple inhabitants. This is a challenging task both for the simulation tool and for the participants. Currently, OpenSHS offers partial support for multiple inhabitants. To increase the realism of the simulations, we plan to integrate VR technologies into OpenSHS in the future. The accessibility for both the researchers and the participants is an important feature. Hence, we plan to port the implementation of OpenSHS to run in a web browser.

## Figures and Tables

**Table 1:** Analysis of smart home simulation tools. OpenSHS, open-source, cross-platform 3D smart home simulator.

Tool/Author(s)	Date	Open-Source	3D	Cross-Platform	Approach	Focus	Multi-Inhabitants	Fast-Forwarding
OpenSHS	2017	Yes	Yes	Yes	Hybrid	Dataset generation	Partially	Yes
Park et al. [32]	2015	No	Yes	Yes	Interactive	Visualisation	No	Yes
PerSim 3D [26]	2015	No	Yes	Yes	Model-based	Dataset generation	No	Not applicable
IE Sim extended [47]	2015	No	No	No	Hybrid	Dataset generation	No	Yes
IE Sim [34]	2014	No	No	No	Interactive	Dataset generation	No	No
Kormányos et al. [48]	2013	No	No	No	Model-based	Visualisation	No	Not applicable
Ariani et al. [35]	2013	No	No	No	Interactive	Dataset generation	Yes	No
Fu et al. [11]	2011	No	No	Yes	Interactive	Visualisation	Yes	No
Jahromi et al. [49]	2011	No	No	No	Model-based	Visualisation	No	Not applicable
Buchmayr et al. [1]	2011	No	No	No	Interactive	Dataset generation	No	No
SimCon [45]	2010	No	Yes	Yes	Interactive	Dataset generation	No	No
YAMAMOTO [42]	2010	No	Yes	Not reported	Interactive	Visualisation	No	No
SIMACT [27]	2010	Yes	Yes	Yes	Model-based	Visualisation	No	Not applicable
Poland et al. [12]	2009	No	Yes	Yes	Interactive	Dataset generation	No	No
ISS [50]	2009	No	No	No	Interactive	Visualisation	Yes	No
DiaSim [29]	2009	No	No	Yes	Model-based	Visualisation	No	Not applicable
V-PlaceSims [38]	2008	No	Yes	No	Interactive	Visualisation	Yes	No
Armac et al. [9]	2007	Not reported	No	Not reported	Interactive	Visualisation	Yes	No
CASS [30]	2007	No	No	No	Model-based	Visualisation	Yes	Not applicable
Krzyska et al. [46]	2006	No	No	Yes	Interactive	Dataset generation	Yes	No
CAST [31]	2006	No	No	No	Model-based	Visualisation	No	Not applicable
UbiREAL [37]	2006	No	No	Yes	Interactive	Visualisation	Yes	No
TATUS [43]	2005	No	Yes	Not reported	Interactive	Visualisation	Yes	No
UbiWise [44]	2002	Yes	Yes	Yes	Interactive	Visualisation	Yes	No

**Table 2:** A set of recorded samples for a particular context.

Samples	Activities				
1	sleep	personal	work	eat	other
2	sleep	personal	other		
3	sleep	personal	other		
4	sleep	eat	personal	other	
5	sleep	eat	personal	other	

**Table 3:** Ten replicated copies based on the samples from Table 2.

i	Activity 1	Activity 2	Activity 3	Activity 4	Activity 5
1	**sample 1** sleep	**sample 1** personal	**sample 1** work	**sample 1** eat	**sample 1** other
2	**sample 4** sleep	**sample 5** eat	**sample 5** personal	**sample 4** other	
3	**sample 3** sleep	**sample 3** personal	**sample 2** other		
4	**sample 3** sleep	**sample 3** personal	**sample 2** other		
5	**sample 5** sleep	**sample 4** eat	**sample 5** personal	**sample 5** other	
6	**sample 1** sleep	**sample 1** personal	**sample 1** work	**sample 1** eat	**sample 1** other
7	**sample 2** sleep	**sample 2** personal	**sample 2** other		
8	**sample 5** sleep	**sample 5** eat	**sample 5** personal	**sample 5** other	
9	**sample 4** sleep	**sample 4** eat	**sample 4** personal	**sample 5** other	
10	**sample 2** sleep	**sample 2** personal	**sample 2** other		

**Table 4:** A sample of the final dataset output.

Timestamp	Bed Table Lamp	Bed	Bathroom Light	Bathroom Door	…	Activity
2016-04-01 08:00:00	0	1	0	0	…	sleep
2016-04-01 08:00:01	0	1	0	0	…	sleep
2016-04-01 08:00:02	0	1	0	0	…	sleep
2016-04-01 08:00:03	0	1	0	0	…	sleep
2016-04-01 08:00:04	1	1	0	0	…	sleep
2016-04-01 08:00:05	1	0	0	0	…	sleep
2016-04-01 08:00:06	1	0	0	1	…	personal
2016-04-01 08:00:07	1	0	0	1	…	personal
2016-04-01 08:00:08	1	0	1	1	…	personal
2016-04-01 08:00:09	1	0	1	1	…	personal
2016-04-01 08:00:10	1	0	1	1	…	personal
⋮	⋮	⋮	⋮	⋮	⋮	

## References

[1] Buchmayr M., Kurschl W., Küng J. (2011). A simulator for generating and visualizing sensor data for ambient intelligence environments. Procedia Comput. Sci..

[2] Rodner T., Litz L. Data-driven generation of rule-based behavior models for an ambient assisted living system. Proceedings of the IEEE Third International Conference on Consumer Electronics.

[3] Youngblood G.M., Cook D.J., Holder L.B. Seamlessly engineering a smart environment. Proceedings of the 2005 IEEE International Conference on Systems, Man and Cybernetics.

[4] Helal S., Lee J.W., Hossain S., Kim E., Hagras H., Cook D. Persim-Simulator for human activities in pervasive spaces. Proceedings of the 7th International Conference on Intelligent Environments (IE).

[5] Tapia E.M., Intille S.S., Larson K. Activity recognition in the home using simple and ubiquitous sensors. Proceedings of the International Conference on Pervasive Computing.

[6] Synnott J., Nugent C., Jeffers P. (2015). Simulation of Smart Home Activity Datasets. Sensors.

[7] Mendez-Vazquez A., Helal A., Cook D. Simulating events to generate synthetic data for pervasive spaces. Proceedings of the Workshop on Developing Shared Home Behavior Datasets to Advance HCI and Ubiquitous Computing Research.

[8] Lei Z., Yue S., Yu C., Yuanchun S. SHSim: An OSGI-based smart home simulator. Proceedings of the 3rd IEEE International Conference on Ubi-media Computing (U-Media).

[9] Armac I., Retkowitz D. Simulation of smart environments. Proceedings of the IEEE International Conference on Pervasive Services.

[10] Helal S., Kim E., Hossain S. Scalable approaches to activity recognition research. Proceedings of the 8th International Conference Pervasive Workshop.

[11] Fu Q., Li P., Chen C., Qi L., Lu Y., Yu C. A configurable context-aware simulator for smart home systems. Proceedings of the 6th International Conference on Pervasive Computing and Applications (ICPCA).

[12] Poland M.P., Nugent C.D., Wang H., Chen L. (2009). Development of a smart home simulator for use as a heuristic tool for management of sensor distribution. Technol. Health Care.

[13] Cook D.J., Youngblood G.M., Heierman E.O., Gopalratnam K., Rao S., Litvin A., Khawaja F. MavHome: An Agent-Based Smart Home. Proceedings of the First IEEE International Conference on Pervasive Computing and Communications.

[14] Alemdar H., Ertan H., Incel O.D., Ersoy C. ARAS human activity datasets in multiple homes with multiple residents. Proceedings of the 2013 7th International Conference on Pervasive Computing Technologies for Healthcare and Workshops.

[15] Munguia Tapia E. (2003). Activity Recognition in the Home Setting Using Simple and Ubiquitous Sensors. Ph.D. Thesis.

[16] Cook D.J., Crandall A.S., Thomas B.L., Krishnan N.C. (2013). CASAS: A smart home in a box. Computer.

[17] WSU CASAS Datasets. http://ailab.wsu.edu/casas/datasets/.

[18] Skubic M., Alexander G., Popescu M., Rantz M., Keller J. (2009). A smart home application to elder-care: Current status and lessons learned. Technol. Health Care.

[19] Nugent C., Mulvenna M., Hong X., Devlin S. (2009). Experiences in the development of a smart lab. Int. J. Biomed. Eng. Technol..

[20] McDonald H., Nugent C., Hallberg J., Finlay D., Moore G., Synnes K. (2013). The homeML suite: Shareable datasets for smart home environments. Health Technol..

[21] Yamazaki T. (2007). The ubiquitous home. Int. J. Smart Home.

[22] Intille S.S., Larson K., Tapia E.M., Beaudin J.S., Kaushik P., Nawyn J., Rockinson R. Using a live-in laboratory for ubiquitous computing research. Proceedings of the International Conference on Pervasive Computing.

[23] PlaceLab Datasets. http://web.mit.edu/cron/group/housen/data/PlaceLab/PlaceLab.htm.

[24] De Ruyter B., Aarts E., Markopoulos P., Ijsselsteijn W. (2005). Ambient intelligence research in homelab: Engineering the user experience. Ambient Intelligence.

[25] Helal S., Mann W., El-Zabadani H., King J., Kaddoura Y., Jansen E. (2005). The gator tech smart house: A programmable pervasive space. Computer.

[26] Lee J.W., Cho S., Liu S., Cho K., Helal S. (2015). Persim 3D: Context-Driven Simulation and Modeling of Human Activities in Smart Spaces. IEEE Trans. Autom. Sci. Eng..

[27] Bouchard K., Ajroud A., Bouchard B., Bouzouane A., Kim T.H., Adeli H. (2010). SIMACT: A 3D Open Source Smart Home Simulator for Activity Recognition. Proceedings of the Advances in Computer Science and Information Technology: AST/UCMA/ISA/ACN 2010 Conferences.

[28] Java Monkey Engine (JME). http://www.jmonkeyengine.com.

[29] Jouve W., Bruneau J., Consel C. DiaSim: A parameterized simulator for pervasive computing applications. Proceedings of the 6th Annual International Mobile and Ubiquitous Systems: Networking & Services.

[30] Park J., Moon M., Hwang S., Yeom K. CASS: A context-aware simulation system for smart home. Proceedings of the 5th ACIS International Conference on Software Engineering Research, Management & Applications (SERA).

[31] Kim I., Park H., Noh B., Lee Y., Lee S., Lee H. Design and implementation of context-awareness simulation toolkit for context learning. Proceedings of the IEEE International Conference on Sensor Networks, Ubiquitous, and Trustworthy Computing (SUTC).

[32] Park B., Min H., Bang G., Ko I. (2015). The User Activity Reasoning Model in a Virtual Living Space Simulator. Int. J. Softw. Eng. Appl..

[33] Unity3D. https://unity3d.com/.

[34] Synnott J., Chen L., Nugent C., Moore G. The creation of simulated activity datasets using a graphical intelligent environment simulation tool. Proceedings of the 2014 36th Annual International Conference of the IEEE Engineering in Medicine and Biology Society (EMBC).

[35] Ariani A., Redmond S.J., Chang D., Lovell N.H. Simulation of a Smart Home Environment. Proceedings of the 2013 3rd International Conference on Instrumentation, Communications, Information Technology, and Biomedical Engineering (ICICI-BME).

[36] Hart P.E., Nilsson N.J., Raphael B. (1968). A formal basis for the heuristic determination of minimum cost paths. IEEE Trans. Syst. Sci. Cybern..

[37] Nishikawa H., Yamamoto S., Tamai M., Nishigaki K., Kitani T., Shibata N., Yasumoto K., Ito M. UbiREAL: Realistic smartspace simulator for systematic testing. Proceedings of the International Conference on Ubiquitous Computing.

[38] Lertlakkhanakul J., Choi J.W., Kim M.Y. (2008). Building data model and simulation platform for spatial interaction management in smart home. Autom. Constr..

[39] FlexSim Software Products, Inc. FlexSim Simulation Software. https://www.flexsim.com/.

[40] Simio, LLC. Simio Simulation Software. http://www.simio.com/.

[41] Rockwell Automation Arena Simulation Software. http://www.arenasimulation.com/.

[42] Stahl C., Schwartz T. Modeling and simulating assistive environments in 3-D with the YAMAMOTO toolkit. Proceedings of the International Conference on Indoor Positioning and Indoor Navigation (IPIN).

[43] O’Neill E., Klepal M., Lewis D., O’Donnell T., O’Sullivan D., Pesch D. A test bed for evaluating human interaction with ubiquitous computing environments. Proceedings of the First International Conference on Testbeds and Research Infrastructures for the Development of Networks and Communities.

[44] Barton J.J., Vijayaraghavan V. (2002). Ubiwise, a ubiquitous wireless infrastructure simulation environment.

[45] McGlinn K., O’Neill E., Gibney A., O’Sullivan D., Lewis D. (2010). SimCon: A Tool to Support Rapid Evaluation of Smart Building Application Design using Context Simulation and Virtual Reality. J. UCS.

[46] Krzyska C. (2006). Smart House Simulation Tool. Ph.D. Thesis.

[47] Lundström J., Synnott J., Järpe E., Nugent C.D. Smart home simulation using avatar control and probabilistic sampling. Proceedings of the 2015 IEEE International Conference on Pervasive Computing and Communication Workshops (PerCom Workshops).

[48] Kormányos B., Pataki B. Multilevel simulation of daily activities: Why and how?. Proceedings of the 2013 IEEE International Conference on Computational Intelligence and Virtual Environments for Measurement Systems and Applications (CIVEMSA).

[49] Jahromi Z.F., Rajabzadeh A., Manashty A.R. (2011). A Multi-Purpose Scenario-based Simulator for Smart House Environments. arXiv.

[50] Van Nguyen T., Kim J.G., Choi D. ISS: The interactive smart home simulator. Proceedings of the 11th International Conference on Advanced Communication Technology (ICACT).

[51] Alshammari N., Alshammari T., Sedky M., Champion J., Bauer C. (2017). Openshs/openshs: First Alpha Release.

[52] (1991). GNU General Public License, Version 2. https://www.gnu.org/licenses/old-licenses/gpl-2.0.en.html.

[53] Blender. https://www.blender.org.

[54] OpenGL. https://www.opengl.org.

[55] Brooke J. (1996). SUS-A quick and dirty usability scale. Usability Eval. Ind..

[56] Katz B.F., Felinto D.Q., Touraine D., Poirier-Quinot D., Bourdot P. BlenderVR: Open-source framework for interactive and immersive VR. Proceedings of the 2015 IEEE Virtual Reality (VR).

[57] WebGL. https://www.khronos.org/webgl/.

